# Over-Expressed miR-224 Promotes the Progression of Cervical Cancer via Targeting RASSF8

**DOI:** 10.1371/journal.pone.0162378

**Published:** 2016-09-14

**Authors:** YongJie Huang, Yang Li, Fen F. Wang, WeiGuo Lv, Xing Xie, Xiaodong Cheng

**Affiliations:** 1 Women’s Reproductive Health Laboratory of Zhejiang Province, Women’s Hospital, School of Medicine, Zhejiang University, Hangzhou, China; 2 Department of Gynecologic Oncology, Women’s Hospital, School of Medicine, Zhejiang University, Hangzhou, China; Beijing Cancer Hospital, CHINA

## Abstract

Cervical cancer is the most common cause of cancer-related deaths in women from developing countries. Identification of novel prognostic predictors or therapeutic targets may improve patient prognosis. In the current study, we demonstrated by real-time PCR that miR-224 expression was significantly upregulated (1.82-fold, P = 0.0025) in cervical cancer tissues (n = 126) compared with in normal cervical tissues (n = 64). Higher expression of miR-224 was significantly associated with poorer prognostic factors, including advanced FIGO stage, nodal metastasis, larger tumor size, vascular involvement and deep stromal invasion (all P < 0.05). Enforced expression of miR-224 promoted cell proliferation, migration and invasion in SiHa and CaSki cancer cell lines. Bioinformatic analysis indicated that RASSF8 (RAS-association domain family 8) was a potential target of miR-224. Western blot analysis and luciferase reporter assay showed that overexpressed miR-224 inhibited RASSF8 protein expression and decreased the activity of a luciferase reporter containing the 3′ untranslated region (UTR) of RASSF8, respectively. Further, RASSF8 knockdown by specific RNAi showed similar effects in cervical cancer cells transfected with miR-224 mimic. Our findings suggest that miR-224 directly targets RASSF8 and thereby acts as a tumor promoter in cervical cancer progression.

## Introduction

Cervical cancer is the third most common cancer in women after breast and colorectal cancer, and contributes to an estimated 530,000 new cases and 275,000 deaths per year [1–3]. Because of a lack of screening and early diagnosis, about 85% of new cases occur in lower socioeconomic areas [4, 5]. Current therapeutics, including surgery, radiation, and chemotherapy, show limited effectiveness for advanced invasive cervical cancer [1]. Cervical cancer remains the most common cause of cancer-related death in women from developing countries. Identifying novel prognostic predictors or therapeutic targets may improve patient prognosis.

MicroRNAs (miRNAs), initially described in 1993 [6, 7], are a group of noncoding small (19–24 nucleotide) RNAs that can either degrade or inhibit translation of target mRNA by binding to its 3' untranslated region (UTR) [8]. Constituting only about 1% of the human genome, miRNAs regulate up to one-third of all genes [9]. Substantial evidence has demonstrated that miRNAs can function as either tumor suppressors or oncogenes; that their deregulated expression could be used as biomarkers for cancer risk, diagnosis, and prognostic prediction; and that they may even be potential therapeutic targets [10, 11].

We previously identified a characteristic miRNA expression profile in cervical cancer by miRNA microarray, in which miR-224 was one of the miRNAs most obviously upregulated in cervical cancer compared with in normal cervical tissues [12]. The role and aberrant expression of miR-224 have been investigated in other cancers, such as hepatocellular carcinoma [13–16], breast cancer [17], lung cancer [18, 19], and colorectal cancer [20]. A previous study also suggested involvement of miR-224 in the development or progression of cervical cancer [21]. In the present study, we confirmed higher expression of miR-224 in cervical cancer tissues than in normal cervical tissues, and demonstrated that miR-224 plays a role in promoting the proliferation, migration and invasion of cervical cancer cells. We also confirmed by bioinformatic analysis [22] and luciferase reporter assay that miR-224 directly targeted RASSF8, a tumor suppressor [23–27], and played an oncogenic role in the progression of cervical cancer. Our findings suggest that miR-224 may have potential clinical applications, for example as a prognostic predictor or therapeutic target in cervical cancer.

## Materials and Methods

### Ethics statement

The use of all tissue specimens was approved by the Hospital Ethics Committee (Permit Number: wydw2009-0010). All the participant provide their written informed consent to participate in the study.

### Patients and tissue specimens

A total of 190 cervical tissue samples, including 126 primary squamous cell carcinoma tissues and 64 normal tissues as controls, was collected. All cervical cancer patients underwent radical hysterectomy with pelvic lymphodenectomy during July 2008 to December 2009 in Women's Hospital, Zhejiang University School of Medicine, and their clinicopathological data were collected. Control samples derived from women who underwent hysterectomy for nonmalignant conditions during the same period. The 75th percentiles of 2^-ΔΔCt^ were used to divide patients into two groups, miR-224 low-expressers (n¼95) and miR-224 high-expressers (n¼31). The correlation between miR-224 expression and clinicopathological parameters was then analyzed in 126 cervical cancer patients. The use of all tissue specimens was approved by the Hospital Ethics Committee. All the histological diagnoses were made by two senior pathologists. All specimens were immediately snap-frozen in liquid nitrogen and stored at −80°C until RNA extraction.

### Total RNA extraction and real-time RT-PCR

Quantitative RT-PCR (qRT-PCR) for miRNA and mRNA was performed after the concentration of total RNA extracted with TRIzol reagent (Invitrogen, Carlsbad, USA) was calculated by measuring absorbance at A260/280. For miRNA quantification, each reverse transcript reaction was set as a final volume of 10 μL consisted of 0.5μg of total RNA, 2.0μL of 5×RT buffer containing dNTPs (Takara Bio, Otsu, Japan), 0.2μL of 10μmol/L stem-loop RT primer (Invitrogen), 0.2 μL RNase inhibitor protein (Takara Bio), and 0.8μL reverse transcriptase (Takara Bio), and incubated at 42°C for 60 minutes at 85°C for 5 minutes. The U6 snRNA was applied as an internal control. Real-time PCR was performed in duplicate with an Applied Biosystems 7900H system (Foster City, CA) using SYBR Premix Ex Taq (Takara Bio). Cycling conditions were 1 cycle of 95°C for 30 seconds and 40 cycles of 95°C for 5 seconds and 60°C for 30 seconds. Total cDNA was synthesized using a TaKaRa reverse transcription kit (Takara Bio). Real-time PCR was performed using SYBR Premix Ex Taq (Takara Bio). EEF1A1 was used as endogenous control for mRNA. All primers as well as the sequences are shown in (Table 1). To determine the relative quantity of miRNA and mRNA expression, the ΔΔCt method was used.

**Table 1:** The sequence of Primers and siR-RASSF8.

Primers and siR-RASSF8	Sequence
miR-224 RT	5'-GTCGTATCCAGTGCAGGTCCGAGGTATTCGCACTGGATACGACTAACCG-3'
miR-224 Forward	5'-GAGCCCAAGTCACTAGTGGT-3'
miR-224 Reverse	5'-GTGCAGGGTCCGAGGT-3'
U6 SnRNA RT	5'-AACGCTTCACGAATTTGCGT-3'
U6 SnRNA Forward	5'-CTCGCTTCGGCAGCACA-3'
U6 SnRNA Reverse	5'-AACGCTTCACGAATTTGCGT-3'
RASSF8 Forward	5'-AGCAGTTCATCCAGCAGACA-3'
RASSF8 Reverse	5'-GAGATGAACCAGGTCGCTTC-3'
EEFIA1 Forward	5'-TGCGGTGGGTGTCATCAAA-3'
EEFIA1 Reverse	5'-AAGAGTGGGGTGGCAGGTATTG-3'
siR-RASSF8-a	5'-GGAUCAACUUCAAGAAAUATT-3'
siR-RASSF8-b	5'-UGACCUUACCGAUCUUUCC-3'

### Cell culture and transfection

SiHa and CaSki cell lines, purchased from the American Type Culture Collection (ATCC, Manassas, VA) and cell bank of Chinese Academy of Science in Shanghai respectively, were cultured at 37°C and 5% CO2 in a humidified incubator in Dulbecco’s modified Eagle’s medium (DMEM) and 1640 medium separately. SiHa derives from local cervical cancer lesion while CaSki is a metastatic cell line Both medium added into 10% fetal bovine serum. Cells were transfected with Dharmacon miRIDIAN miR-224 mimic (Thermo Fisher Scientific, Lafayette, CO),siRNA(Ribobio,Guangzhou,China) and their corresponding negative control (miR-NC,siRNA ctrl) at a final concentration of 50 nmol/L, using DharmaFECT1 transfection reagent (Thermo Fisher Scientific) according to the manufacturer's instructions.

### Cell proliferation analysis

To find out the influence of miR-224 on proliferation of cervical cell lines, 96-well plates were applied to culture SiHa (5×10^3^ cells per well) and CaSki (4×10^3^ cells per well) cells overnight. The cell viability was assessed by MTT assay at 0, 48 and 96 hours after transfection. 4 hours before incubation stopped, 20μL of MTT solution was added into each well. The absorbance of samples was measured with a spectrophotometer reader (Elx800, Bio-TEK, Winooski, VT, USA) at a wavelength of 490 nm after the supernatant removed and 150 μL of dimethylsulfoxide (DMSO) was added. All experiments were conducted in six replicates and were repeated three times independently. Data are presented as means **±** SD of three separate experiments.

### Cell cycle and apoptosis analysis

For apoptosis assays, adherent cells were removed by trypsin enzyme and washed by phosphate-buffered saline (PBS) at 48h after transfecion. Annexin-V and propidium iodide (Biouniquer) was added to the cells successively.30min after being stained, flow cytometry was used to detected and analyze the fluorescence of the sample (Beckman Coulter, Fullerton,CA, USA). For cell-cycle analysis, 48 h after transfection, 10 ml of 70% ethanol solution was directly added into 1ml of suspended cervical cancer cells. The treated cells were then fixed for (48 h) before staining. The following staining protocol was performed according to the manufacturer’s guidelines (BD Pharmingen BrdU Flow Kit, BD Biosciences, San Jose, CA, USA). Finally, flow cytometry was used to analyse the fluorescence of the sample (Beckman Coulter, Fullerton,CA, USA).

### Cell migration and invasion assay

To assess ability of tumor cell motility, an in vitro wound-healing assay was performed. SiHa cells (4×10^5^/well) and CaSki cells (4 ×10^5^/well) were seeded in12-well plates and transfected with miR-224 mimic. When the culture had reached nearly 90% confluency, the monolayer was scratched with a 10μl sterile Plastic pipette tip and PBS was used to wash the cellular debris. Cells were cultured again in opti-MEM medium at 37°C in a humidified incubator with 5% CO_2_. At different time points, photographic images of the plates were taken. Wound healing was measured at 0, 24, and 48 hours, and the data were summarized based on six assays for each experiment.

Invasion assay was performed in transwell chambers with members coated with CultrexBasement Membrane Extract without Phenol Red (R&DSystems, Minneapolis, MN). Cells(2×10^4^/well) transfected with miR-224 mimic or siR-RASSF8 were suspended in 200 μL medium without serum and seeded on the upper chamber. the lower chamber was filled with 10% fetal bovine serum. After 48 hours, cells on the upper side of the membrane were removed andthose on the lower side of the membrane were fixed and stained with crystal violet solution. The cells under the microscopic fields (100×objective) in each chamber were photographed and counted; All invasion assays were done in triplicate for at least three independent experiments.

### Luciferase report assay

The pmirGLO dual-luciferase miRNA target expression vector (pmirGLO vector) containing both firefly luciferase gene and Renilla luciferase gene was purchased from Promega (Madison, WI). Human RASSF8 3'-UTR including the predicted binding site of miR-224 was inserted into the 3'-UTR region downstream of the firefly luciferase gene of pmirGLO vector (pmirGLO-UTR). A site-directed gene mutagenesis kit (Beyotime, Jiangsu, China) was used to construct the mutant type of miR-224-binding sites vector (pmirGLO-mUTR) according to the manufacturer's protocol. Cotransfection of miRNA mimics (50nmol/L) and reporter vectors (0.2μg/mL) was performed using invitrogen lipofectamine 2000 transfection reagent. Luciferase activities were measured at 24 hours after transfection using a Dual-Glo^™^ Luciferase Assay kit (Promega), and firefly luciferase activities were normalized to Renilla luciferase activities. Experiments were performed in triplicate and repeated twice. Data are presented as means± SD.

### Western Blot Analysis and IHC

72 hours after transfection with miR-224 mimic, total protein was extracted from the cells. The primary antibodies used for Western blot analysis as well as IHC were anti-RASSF8 antibody (1:2000) and (1:100) obtained from Santa Cruz Biotechnology (Santa Cruz, CA). Anti-GAPDH antibody as endogenous control (1:2000) was also purchased from Santa Cruz Biotechnology (Santa Cruz, CA). Secondary antibodies (1:2000) were purchased from Dawen Biotec (Zhejiang, China) and Dako (Dako Diagnostica, Hamburg, Germany) for western blot and IHC respectively.

### Statistical analysis

Data are presented as means _ SD from at least three independent experiments. Student’s t-test was used to analyze differences of the result in experiments with cell lines. Correlation between expression levels of miR-224 and its target genes in cervical cancer tissues was analyzed using Pearson’s correlation coefficient. Association between expression level of miR-224 and each clinicopathologic parameter was evaluated using Pearson’s _2 test. All statistical tests were two-sided, and P values of _0.05 were considered statistically significant. All analyses were performed using SPSS 16.0software(SPSS,Chicago,IL).

## Results

### The level of miR-224 is upregulated in cervical cancer tissues and associated with clinicopathological characters of the patients

**Fig 1:**
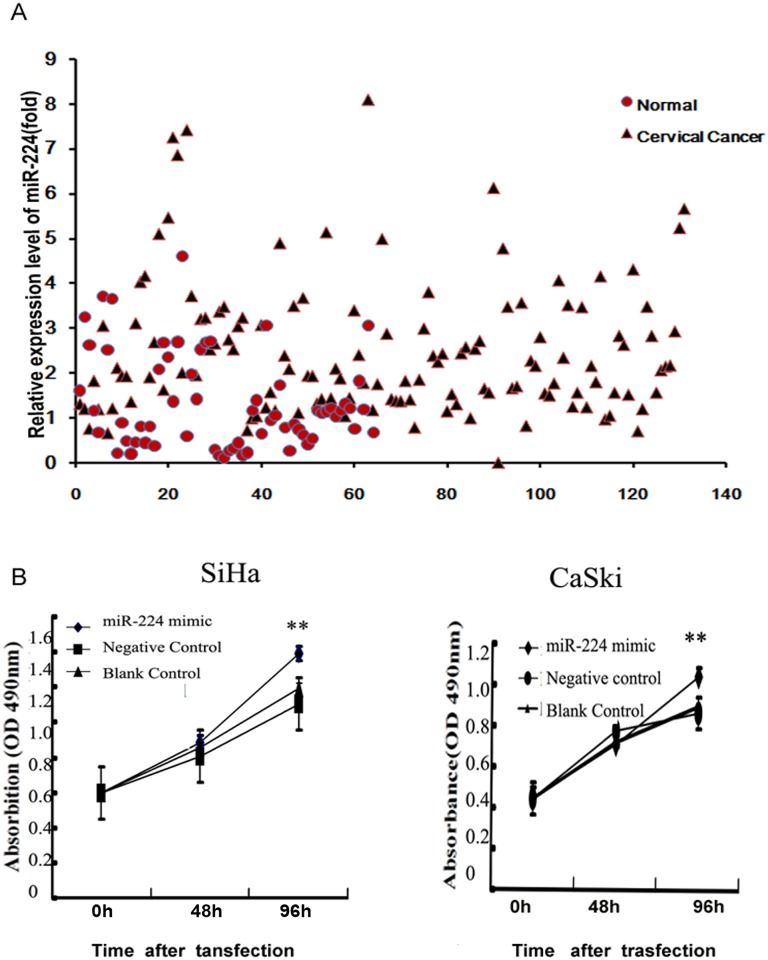
miR-224 expression was higher in cervical cancer tissues. miR-224 over-expression enhanced cell proliferation. (A) miR-224 expression was higher in cervical cancer tissues compared with that in cervical normal tissues by real-time PCR analysis. (B) At 0, 48, and 96h after transfection, cell proliferation was measured by the MTT assay. Data were presented as mean±s.e. from three independent experiments.

In our previous miRNA microarray, the expression level of miR-224 in cervical cancer tissues was raised to 2.32-fold compared with that in normal cervical tissues[12]. In our present study, the expression level of miR-224 was raised by 1.82-fold (P = 0.0025) in cervical cancer tissues compared with that in normal tissues (Fig 1A). Correlation between miR-224 expression level and clinicopathological characters of cervical cancer was summarized in Table 2. The miR-224 high-expressers were significantly associated with advanced FIGO stage, nodal metastasis, larger tumor size, vascular involvement and deep stromal invasion (all P<0.05), suggesting that higher expression level of miR-224 may be involved in the progression and metastasis of cervical cancer.

**Table 2:** Association between miR-224 expression and clinicopathological parameters of cervical cancer.

Clinicopathological parameters	Total(n = 126)	miR-224 expression		χ²	*P* value
		high	low		
		31	95		
**Age, years**					
**≦35**	11	3	8	0.046	0.83
**≧35**	115	28	87		
**FIGO stage**					
**I**	86	16	70	5.255	**0.027**
**II**	40	15	25		
**Pathological grade**					
**Grade1**	3	1	2	2.91	0.127
**Grade2**	105	22	83		
**Grade3**	18	8	10		
**Tumor size**					
**≤4**	106	22	84	9.268	**0.005**
**>4**	20	9	11		
**Lymph node metastasis**					
**Negative**	116	25	91	5.006	**0.04**
**Positive**	10	6	4		
**Vascular involvement**					
**Negative**	101	21	80	4.653	**0.038**
**Positive**	25	10	15		
**Deep stromal invasion**					
**<66%**	85	16	69	5.255	**0.027**
**≧66%**	41	15	26		
**vaginal wall extention**					
**yes**	14	5	9	1.048	0.33
**no**	112	26	86		
**parametrail extention**					
**yes**	6	2	4	0.063	0.83
**no**	120	29	91		

FIGO: International Federation of Gynecology and Obstetrics

### miR-224 enhances cell proliferation in cervical cancer cell lines

SiHa and CaSki were transfected with miR-224 mimic or miR-NC (miRNA negative control). Cell proliferation was measured at 0h, 48h and 96h after transfected with miR-224 mimic by MTT essay. The growth curve showed a significant increase of cell viability at 96h in both cell lines (SiHa, 96h, p = 1.8278E-08; CaSki, 96h, p = 0.0011; Fig 1B). Cell apoptosis and cell cycle assay were carried out at 48h after transfection of miR-224 mimic in both cell lines (SiHa and CaSki), but no significant differences between them were observed. Thus, our results suggest that over-expressed miR-224 accelerates cell proliferation but cell apoptosis or cell cycle.

### Over-expressed miR-224 promotes migration and invasion in cervical cancer cells

**Fig 2:**
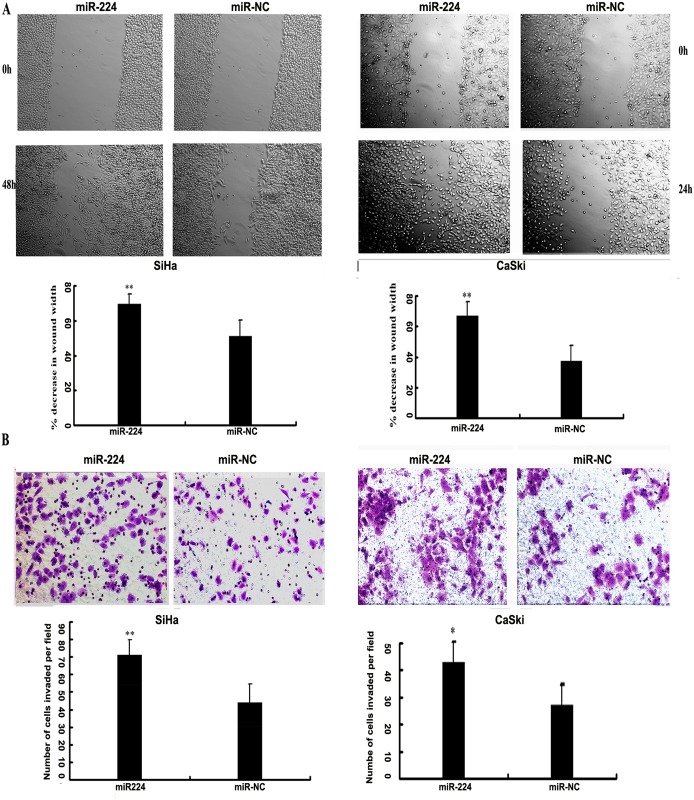
miR-224 promoted the migration and invasion in cervical cancer cells. (A) Wound healing assay was implemented in cervical cancer cell line SiHa and CaSki after transfected with 50 nM of miR-224 mimic or negative control (miR-NC). The wound healing was measured at the time points as indicated. Bars represented the average percentage of wound healing ±s.d. ** P<0.01. (B) Cell invasion capabilities were measured in SiHa and CaSki cells with transwell chambers, as described in materials and methods. Photos were representative fields of invasive cells on the membrane with a 200-fold magnification. Bar graphs represented the average number of cells per field on the underside of the membrane ±s.d. ** P<0.01.

The wound-healing assay and transwell invasion assay were conducted to further confirm the function of miR-224 on the progress of cervical cancer. Wound healing assay showed that over-expression of miR-224 was more efficient compared to miR-NC in closing an artificial wound (SiHa, 48h, P = 1.8561E-08; CaSki, 24h, p = 3.9037E-13; Fig 2A). In addition, raised miR-224 expression significantly enhanced the invasive capacity of both cancer cell lines by matrigel chamber assays (SiHa, P = 0.035; CaSki, P = 0.0004; Fig 2B). Therefore, our results revealed that miR-224 served as a promoter on invasion and metastasis in cervical cancer cells, thus contributing to the progression of cervical cancer.

### RASSF8 serves as direct target of miR-224 in cervical cancer cells

**Fig 3:**
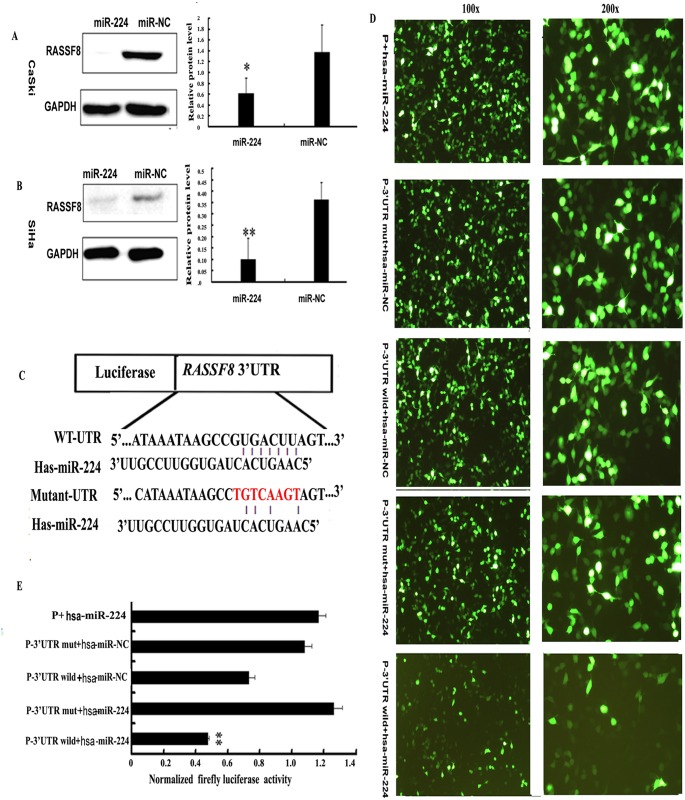
miR-224 suppressed RASSF8 expression and directly targeted 3'UTR of RASSF8 mRNA. (A,B) At 72h after transfected with 100 nM of miRNA mimic and negative control, the endogenous protein levels of RASSF8 in SiHa and CaSki cells were measured by western blot. Bars indicated the relative protein levels that were normalized to GAPDH. Data were presented as mean±s.d. (n¼3) *P<0.05, **P<0.01. (C) Putative miR-224-binding site in the RASSF83'-UTR mutation was generated by mutating 3nt that is recognized by miR-224. Either wild-type (WT) or mutant RASSF8 3'-UTR was subcloned into the dual-luciferase reporter vector. (D) The luciferase reporter vector containing wild (wild type) RASSF8 3’-UTR or mutant RASSF8 3’-UTR was cotransfected into SiHa cells with miR-224 mimic or miRNA negative control. (E) Firefly luciferase activities were determined at 48h postransfection and normalized to Renilla luciferase. Each column represented the mean±s.d. of three independent experiments. **P <0.01.

We searched public algorithms (TargetScan, PicTar, and microbase)[28] [22]for candidate target genes mediating the observed effects of miR-224. Among those, RASSF8 was selected for it serves as a tumor suppresser in lung cancer. Western blot analysis showed that the quantity of RASSF8 protein expressive level of SiHa and CaSki cells transfected with miR-224 mimic was significantly decreased (Fig 3A and 3B). Further, dual-luciferase report assay was performed. The reporter vector, carrying partial sequence of the 3'untranslated region (3'-UTR) of RASSF8, was then cotransfected with miR-224 mimic. Compared with the negative control a significant decline of luciferase activity was observed with miR-224 transfection (P = 5.5538E-05; Fig 3D). Moreover, mutation of the predicted-binding site of miR-224 on the RASSF8 3'-UTR reactivated the luciferase (P = 3.3768E-06; Fig 3D and 3E). Thus we validated the direct interaction between miR-224and RASSF8.

### RASSF8 expression was decreased in cervical cancer tissues

**Fig 4:**
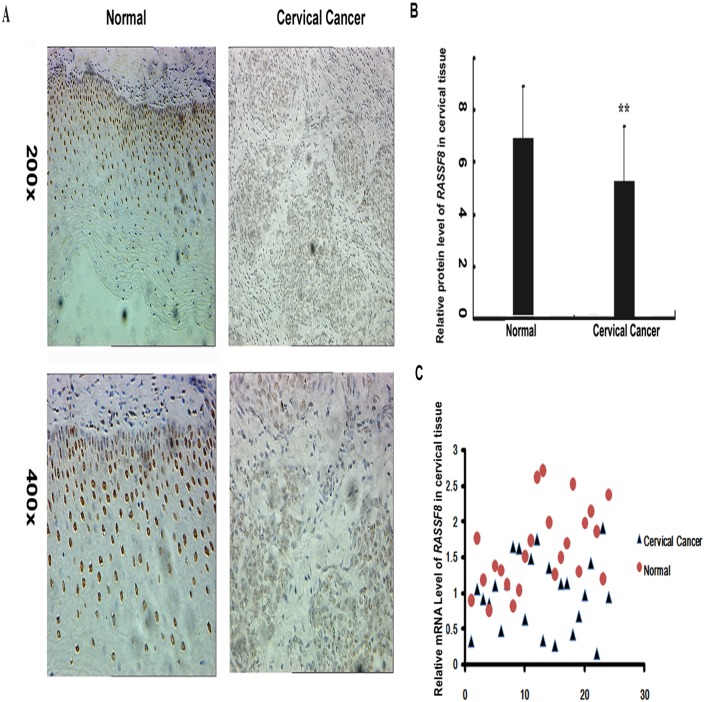
Protein and mRNA levels of RASSF8 were decreased in cervical cancer tissues. (A) Protein level of RASSF8 in normal cervical epithelium and cervical cancer specimens was measured by IHC. The positive staining of RASSF8 was in the nuclear and more strongly in normal cervical epithelium. The expression levels of RASSF8 were determined by assessing its staining using software image pro-plus 6.0. The results were showed as integrated optical density (IOD)/area. Bar represented the average values of corresponding protein levels in normal cervical epithelium and cancer tumors ±s.e. (B) Real-time PCR validated the mRNA levels of RASSF8 in cervical cancer tissues compared with tht in normal cervical tissues. Mean ± s.e. **P<0.01.

We examined the protein and mRNA levels of RASSF8 expression in cervical tissues by immunohistochemistry and Realtime PCR respectively in cervical cancer(n = 25) and normal tissues(n = 25). As illustrated in Fig 4, both IHC staining and qRT-PCR showed a visible fall of RASSF8 expression levels in cervical cancer tissues with average value of 0.77 and 0.65 compared with that in the normal tissue as 1.0, respectively (P = 0.0018, Fig 4A and 4B; P = 0.0218, Fig 4C). Thus our in vivo experiments confirm that RASSF8 serve as a target of miR-224 participated in tumor progression in cervical cancer.

### RASSF8 participates in multiple biological processes in cervical cancer cells

**Fig 5:**
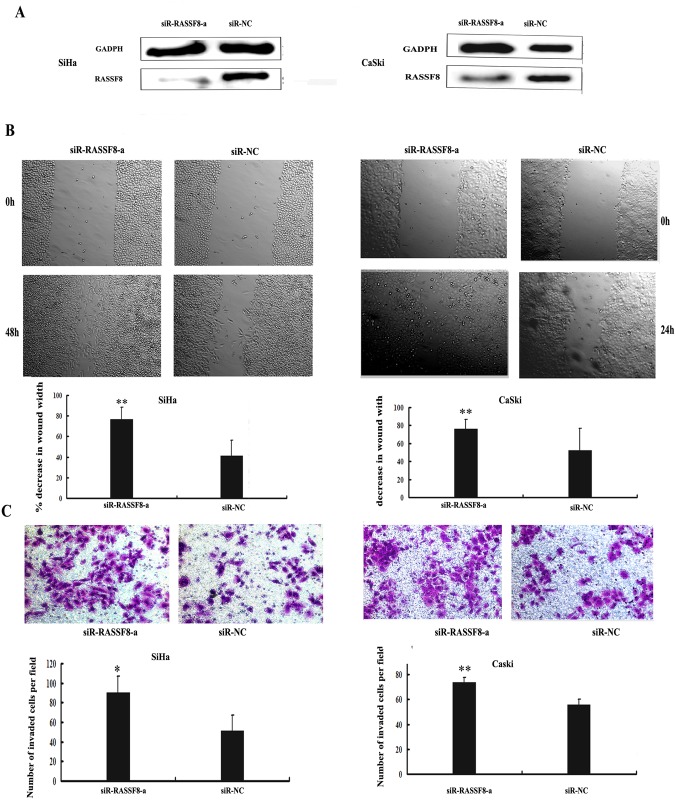
siR- RASSF8-a inhanced cell migration and invasion in SiHa and CaSki cells. (A) Specific siRNA targeting RASSF8 suppressed RASSF8 protein expression. SiHa and CaSki cells were transfected with 50 nmol of siRNA RASSF8 or siRNA-negative control (siR-NC) and the transfection efficiency were assessed. RASSF8 protein expression levels were determined by western blot analysis. GAPDH was served as the internal control. (B)Representative images of wound healing were taken at 0h, 24h, and 48h after the wound scratched. Data are presented as mean±s.d. *P<0.05, **P<0.01. (C) The number of invaded cells following RASSF8 knockdown using specific siRNA targeting RASSF8(siR-RASSF8-a) versus negative control (siR-NC) were shown by stained with crystal violet. Bars indicated the invasion capability compared with that of negative control ± s.d*P<0.05, **P<0.01.

**Fig 6:**
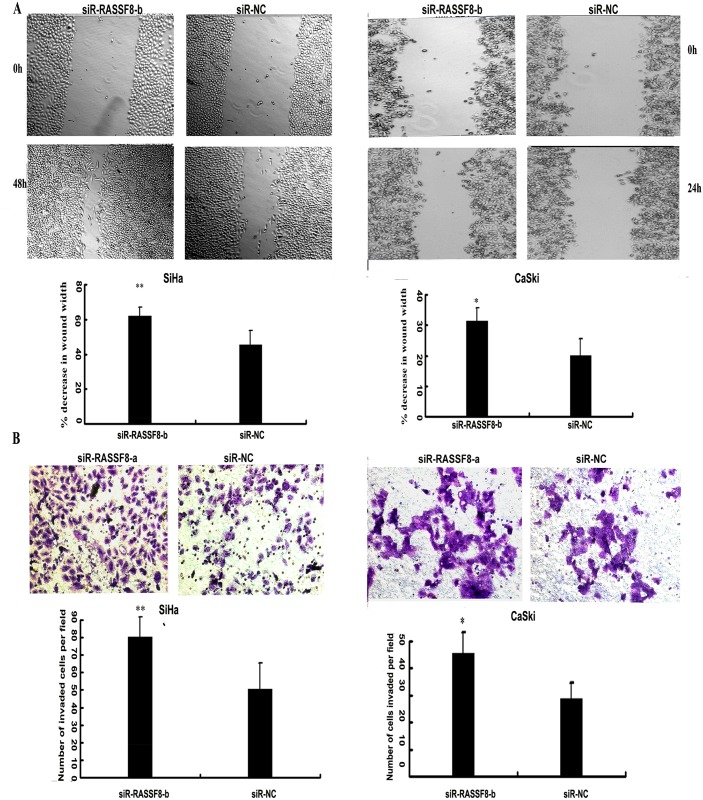
siR-RASSF8-b inhanced cell migration and invasion in SiHa and CaSki cells. A)Representative images of wound healing were taken at 0h, 24h, and 48h after the wound scratched. Data are presented as mean±s.d. *P<0.05, **P<0.01. B) The number of invaded cells following RASSF8 knockdown using specific siRNA targeting RASSF8 (siR-RASSF8-b)versus negative control (siR-NC) were shown by stained with crystal violet. Bars indicated the invasion capability compared with that of negative control ± s.d*P<0.05, **P<0.01.

To ascertain that RASSF8 serves as a critical mediator of miR-224's biologic role in cervical cancer cells, siR-RASSF8-a and siR-RASSF8-bspecific for knocking down RASSF8 mRNA were then synthesized. The sequence of the two siR-RASSF8s are shown in Table 1. Western blot analysisshowed that siR-RASSF8-a reduced RASSF8 protein levels by 2-fold in both cell line SiHa and CaSki as showed in Fig 5A. Real-time PCR showed that siR-RASSF8-b reduced RASSF8 protein levels by 2.5-fold in both cell line SiHa and CaSki as showed in S2 Fig. In accordance with what was observed by miR-224 overexpression, siR-RASSF8-a promoted the migratory ability (SiHa, 48h, P = 6.5451E-05; CaSki, 24h, P = 2.4633E-05, respectively; Fig 5B) and the invasive capability (SiHa, P = 0.0203; CaSki, P = 0.0035; Fig 5C) of both cell lines. siR-RASSF8-b further confimed the result by enhancing the migratory ability (SiHa, 48h, P = 0.00635; CaSki, 24h, P = 0.044, respectively; Fig 6A) and the invasive capability (SiHa, P = 0.00703; CaSki, P = 0.035; Fig 6B) of both cell lines. Taken these results together, RASSF8 negatively regulated by miR-224 serves as a direct target of miR-224 in progression and metastasis of cervical cancer cells.

## Discussion

miR-224, which is located on the X-chromosome [29–31], is active in mammalian ovaries [29]. Recently, miR-224 was reported to be aberrantly expressed in various human malignancies and associated with cancer development. However, like other miRNAs, miR-224 displays different expression modes in different cancers. For instance, miR-224 is upregulated and acts as a promoter in liver cancer [13–16], breast cancer cells [17], and lung cancer [18, 19]. Conversely, miR-224 is down-regulated in methotrexate resistant colon cancer cells [32] and prostate cancer [33]. We previously conducted a miRNA microarray to explore specific miRNA expression profiles and identify a specific miRNA that participates in the development of cervical cancer, and which could therefore provide a potential biomarker for predicting prognosis or even be a target for cervical cancer therapy. We observed markedly higher miR-224 expression levels in cervical cancer tissues than in normal cervical tissues [12]. The clinical significance of miR-224 during cervical cancer development was confirmed by our findings and by the results of other studies, which indicated that cervical cancer patients with higher levels of miR-224 tend to have poorer prognosis [21]. However, the biological function of miR-224 and the mechanisms by which it is involved in cervical cancer initiation or progression remain unclear. In the present study, we demonstrated by real-time PCR that miR-224 expression was increased by 1.82-fold in cervical cancer tissues compared with in normal cervical tissues. Further, we showed that elevated levels of miR-224 expression were associated with clinicopathologic characteristics linked to poorer prognosis, suggesting that miR-224 likely promotes the progression of cervical cancer. To support our clinical findings, we overexpressed miR-224 in cervical cancer cells by miR-224 mimic transfection and observed the effect on cell biological behaviors. We found that raised miR-224 expression promoted proliferation, and enhanced migration and invasion in SiHa and CaSki cells. Thus, our *in vivo* and *in vitro* findings together suggest that miR-224 acts as a promoter for tumor progression in cervical cancer and may have potential application as a predictor of prognosis in cervical cancer patients.

To gain a further insight into the biological functions of miR-224 in cervical cancer cells, we identified its target genes. Using the available computational approaches, we predicted target genes for miR-224 [22, 28]. Among various predicted cancer-associated genes, we selected RASSF8 as a candidate target for further study. The RAS-association domain family (RASSF) comprises ten member proteins, which are potential tumor suppressors. They all are characterized by the inclusion of an RA-domain (RAS-association domain) at either their C-terminus (RASSF1–6) or N-terminus (RASSF7–10) [23, 25]. RASSF proteins are linked to key biological processes, including cell death, proliferation, microtubule stability, promoter methylation, vesicle trafficking, and response to hypoxia [25, 27]. RASSF8, an N-terminus RASSF, has been shown to be ubiquitously expressed throughout the murine embryo and in normal tissues of human adults [34], and has been described as a potential tumor suppressor in lung carcinogenesis [26, 35, 36]. In this study, we demonstrated an upregulation of miR-224 and a downregulation of RASSF8 in cervical cancer tissues. Furthermore, we showed that levels of RASSF8 protein expression were significantly downregulated by overexpressed miR-224; and we identified the existence of a binding site for miR-224 in the RASSF8 3^**'**^ UTR by luciferase activity assay. Additionally, RASSF8 knockdown using specific siRNA enhanced migration and invasion of SiHa and CaSki cells. Thus, we confirmed that RASSF8 is a direct target for miR-224 and mediates miR-224 promotion of cervical cancer progression.

## Conclusion

Taken together, our results demonstrate that miR-224 expression is increased in cervical cancer tissues compared with in normal tissues, and that raised miR-224 expression is linked to poorer prognosis in cervical cancer patients. By directly targeting RASSF8 and inhibiting RASSF8 expression, upregulated miR-224 enhances proliferation, migration, and invasion of cervical cancer cells, and consequently promotes the progression of cervical cancer. Our findings suggest that miR-224 acts as a tumor promoter in cervical cancer, and that miR-224 and its target RASSF8 protein have potential for use as prognostic predictors or therapeutic targets in cervical cancer patients.

## Supporting Information

S1 Fig miR-224 and RASSF8 expression level in both cell lines. Data are presented as fold of the internal control. GAPDH and U6 SnRNA were served as internal control for RASSF8 and miR-224 respectively.
(TIF)

S2 Fig siR-RASSF8-b suppressed RASSF8 protein expression. Specific siRNA targeting RASSF8 suppressed RASSF8 miRNA expression. SiHa and CaSki cells were transfected with 50 nmol of siRNA RASSF8(siR-RASSF8-b) and siRNA-negative control (siR-NC) and the transfection efficiency were assessed. RASSF8 miRNA expression levels were determined by Real-time PCR. GAPDH was served as the internal control.
(TIF)

S3 Fig The full uncropped raw blots for the Western blot results. (TIF)

S1 Table The underlying data points of both cervical cancer cells transfected with siR-RASSF8. (XLS)

S2 Table The underlying data points for the result of CaSki cells trasnsfected with miR-224. (XLS)

S3 Table The underlying data points for the result of Wounding-healing and Traswell of Siha cells transfected with miR-224 mimic. (XLS)

S4 Table The underlying data points for the MTT result of Siha cells transfected with miR-224 mimic. (XLS)

S5 Table The underlying data points for the Western blot result of Siha cells transfected with miR-224 mimic. (XLS)

S6 Table The underlying data points for immuntochemistry(ICH). (XLS)

S7 Table The underlying data points for Luciferase report assay. (XLSX)

S8 Table The expression level of miR-224 in cervical tissue. (XLSX)

S9 Table The mRNA level of RASSF8 in cervical tissue. (XLSX)
