# Supplementary figures and images for: Over-Expressed miR-224 Promotes the Progression of Cervical Cancer via Targeting RASSF8
Source: PLoS One. 2016 Sep 14;11(9):e0162378. doi: 10.1371/journal.pone.0162378 (PMC5023165; doi:10.1371/journal.pone.0162378)

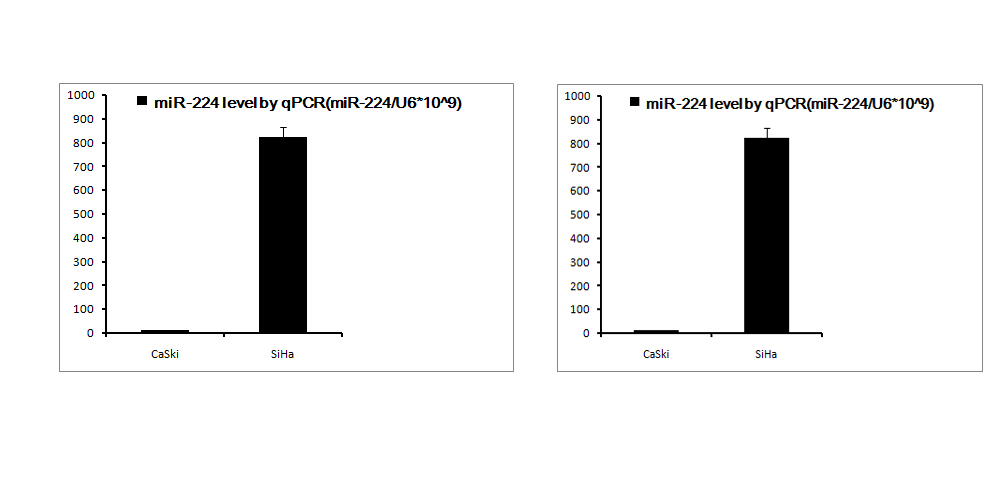

Supplement: S1 Fig — Data are presented as fold of the internal control. GAPDH and U6 SnRNA were served as internal control for RASSF8 and miR-224 respectively. (TIF) [file pone.0162378.s001.tif]

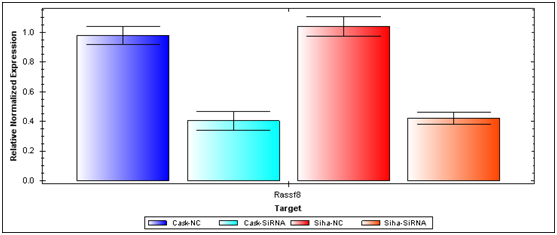

Supplement: S2 Fig — Specific siRNA targeting RASSF8 suppressed RASSF8 miRNA expression. SiHa and CaSki cells were transfected with 50 nmol of siRNA RASSF8(siR-RASSF8-b) and siRNA-negative control (siR-NC) and the transfection efficiency were assessed. RASSF8 miRNA expression levels were determined by Real-time PCR. GAPDH was served as the internal control. (TIF) [file pone.0162378.s002.tif]

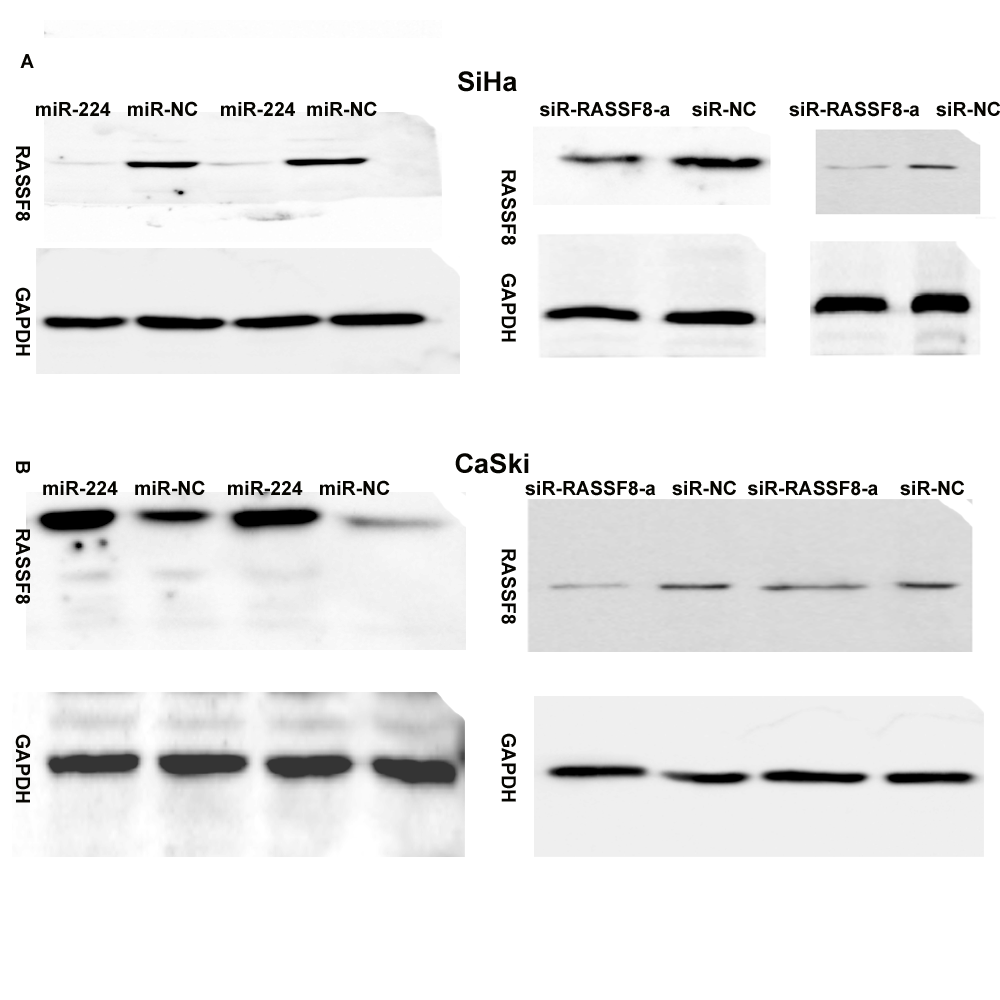

Supplement: S3 Fig — (TIF) [file pone.0162378.s003.tif]
